# "The Hospital" Medical Book Supplement, No. 2

**Published:** 1907-12-21

**Authors:** 


### "THE HOSPITAL" MEDICAL BOOK SUPPLEMENT.—No. II.

CONTENTS.

**The Practitioner's Library** - - - - - - - - 319

**Medicine—**
The Re-Education of Co-Ordination by Movements - - - 320
Some of the Clinical Aspects of Pneumonia - - - - 321
Hygiene of Nerves and Mind in Health and Disease - - - 321
The Therapeutical Society's Transactions - - - - - 321

**Books Received** - - - - - - - - - - 322

**Surgery—**
Some Points in the Surgical Anatomy of the Temporal Bone from Birth to Adult Life - - - - - - - - - 321
Surgical Applied Anatomy - - - - - - - - 321
Diseases of the Male Generative Organs - - - - - - 322

**Special Subjects—**
The Bacteriological Examination of Disinfectants - - - 322
Text-book of Organic Chemistry for Medical Students - - 322

### THE PRACTITIONER'S LIBRARY.

When the student has finished his course and embarks on private practice he has already formed, in the great majority of cases, the nucleus of a library. The value of such a student collection, from the practitioner's point of view, varies greatly. The student who has been reading for examinations merely will have acquired a small collection of familiar text-books, rich in theory, but for the most part lacking in practical utility. Modern students' text-books usually claim that they have special advantages for the general practitioner as well; it is not uncommon to see, on the title-page of some examination cram book, the announcement that it is a volume "destined for the use of students and practitioners." For the most part such a claim is exaggerated, to say the least. The pathology, the morbid anatomy, the theories of causation, and the hypotheses of various out-of-the-way signs and symptoms—points which some books dwell on to the exclusion of the major part of treatment, prophylaxis and pure symptomatology—are of but little use to the man in general practice, and treatment, unfortunately, is a subject which most examination text-books deal with in a very perfunctory fashion. The acquisition of half a dozen standard text-books is to be commended, but the commendation should be conditioned by the suggestion that the practitioner's library needs something more full, more extended, more catholic than these.

Away from his teachers and hospital reference library, the man who has newly embarked upon private practice feels the want, more especially, of sound up-to-date text-books on the "specialties." His library, therefore, should include at least one good volume on each of the systems to which it has become customary to devote special volumes: the nose, the ear, the throat, the eye, the nervous system, and so on. The ordinary text-books will tell him little that he does not know of the more familiar disorders he may meet with. What they have been able to teach him he should have learned during his student days; they can only serve as reference books, to be picked up, hastily conned, and then replaced. But the medical man does not cease to be a student the moment he has obtained his diploma; he remains one all his life, learning the major part of his work from actual experience. There are some subjects with which he cannot get an adequate acquaintance first hand; he must obtain his information about them from the literature, and for that reason it is imperative that his library should be a growing one. The old favourites, the books he loved in his student days, which he trusts in practice will remain helpful to the last, will need to be supplemented by other volumes as the subjects with which they deal become better known and more correctly understood. The acquisition of a new edition of an old favourite is not always sufficient to give the practitioner the best insight into the latest theories, and the custom which is observed by some of the older practitioners, of annotating their old editions, is much more to be commended than the indiscriminate multiplication of new editions upon the library shelves. It is rarely indeed that a new edition proves eminently satisfactory to one who loved and relished the old, and one of the most recent new editions is a striking example of the manner in which an old favourite may be spoiled by injudicious enlargement without discrimination.

We have already said that the practitioner's library should be catholic. It should include more than mere text-books or monographs on medicine, surgery, and midwifery. And here we would put in a special plea for the acquisition of editions of classical writers, now so unfortunately neglected by medical men. The excellent series of translations which the enthusiasm of the New Sydenham Society has produced should be known to every practitioner, and one or two, at least, of the better-known classical writers on medicine should find a place on the library shelves. There exists, unfortunately, no good exposition of the history of medicine in English; the old editions of Freind and others are no longer procurable, and many of the recent books dealing with the subject are of more special than general interest; but we would plead for the inclusion of some standard work on the history of medicine, even if the student has to resort to a German dictionary to read it.

There is no sharp line of demarcation between the professional and the non-professional library, and the man who is likely to make the most use of his books is probably he who will not attempt to marshal purely technical volumes on one shelf and books dealing with cognate subjects on another. We do not think any general practitioner, not specially interested in medical literature, will concentrate his energies on the formation of a large library. It is not the number of volumes that counts; rather is it their usefulness; and with usefulness almost necessarily goes interest. The outlay upon a series of monographs, such as one of the various excellent "Systems" of medicine and surgery, will be more than repaid in the end by the benefits, both practical and intellectual, which the purchaser will derive from the volumes. The little extra time expended in obtaining a reading knowledge of French or German or Italian will equally be well repaid by the added interest which the practitioner will derive from reading books in these languages, and there are certain standard works of Continental writers which should find a place on the library shelves. A small, well-chosen collection of good books, not ephemeral trash that requires re-editing every year, must be the ideal that the general practitioner should strive for, and in the selection of his books individual taste must largely be the controlling factor.

For that reason we do not purpose giving a list of the books which, in our opinion, are suitable for inclusion in the ideal library. Such lists can, no doubt, be drawn up, and to many, as we know from inquiries which at various times are made as to advice in the selection of books, they may be of use. But the best library is the result of careful thought and as careful selection. There is no book always acceptable, as Milton has pointed out, and variety in reading, as in everything else, brings its reward in added pleasure and enhanced profit. The mere buying of a professional work because it has been well reviewed or is well spoken of, is hardly to be recommended, for it is not by such methods that the ideal library is collected. Rather by carefully choosing each work on its own merits, apart altogether from what others say of it, can the practitioner acquire a collection that will be of lasting use and a source of continual pleasurable reading to him. "Buy books that have stood the test of time" is a limitation of choice which we do not care to endorse, but it contains the kernel of sage counsel in so far as it advises earnest consideration before a selection is made. To take up a publisher's catalogue and tick off certain volumes, hall-marked perhaps by the fact that they bear on their title-pages the names of men who are authorities in their special subjects, is an easy method—to the man who can afford it—of acquiring a well-stocked professional library. Whether it is a method which will enable him to form a useful and really efficient collection is another question altogether, and one which we are inclined to answer in the negative.

A corner of the practitioner's library must be devoted to periodical publications. No practitioner can afford to be without his weekly or monthly journal, which keeps him alive to the constant changes which are proceeding in his profession, in touch with new movements of thought, and makes him sensible of the great alterations which are going on in revision and emendation, which he is otherwise liable to forget. These periodicals need not be ephemeræ, and should certainly not be treated as such. At the end of their year they should be collected and bound, and the bound volumes should be kept on a shelf, ready for reference. For to a great extent the practitioner's library should be essentially a library of reference books, to which he can refer when he wishes to "look up" a case, a theory, a line of treatment, an operation, a diagnosis, a point in pathology, or a question of prophylaxis. And to be that, in the fullest sense, it should be catholic, embracing all the various branches of medicine and the cognate sciences.

### MEDICINE.

The Re-Education of Co-Ordination by Movements, with Special Reference to Locomotor Ataxy. By A. G. Dampier-Bennett, M.R.C.S., L.R.C.P. Pp. 15. (Published by John Wright and Company, Bristol. Price, together with Mounted Charts for the Movement Exercises, 10s. 6d. net.)

There is not a great deal to read in this small booklet, but the subject dealt with is important. Great Britain is a long way behind the Continent in the matter of treatment of tabes dorsalis and other ataxic conditions by suitable co-ordinating exercises; and the results obtained abroad have proved convincingly that, by very simple measures, it is possible to postpone the severer stages of ataxy for years longer than is the case when such measures are not resorted to. The essential principles of the treatment are mainly three, namely :—

1. That the exercises should be as simple as possible, entailing as little muscular effort as may be, whilst calling for as much purposeful co-ordination of muscles as possible. The object of the exercises is not to increase the muscle strength or bulk, but to give practice in causing the existing muscles to act together co-ordinately.

2. That the exercises should be, as far as possible, different from those called for repeatedly during the patient's daily avocation.

3. That the practice should be for comparatively short periods at a time, ceasing invariably before fatigue is felt, but repeated daily with regularity, with such simple variations as may be indicated from time to time.

We feel that this method of dealing with ataxy should be more widely known and adopted in this country, and to this extent we are glad to see this publication. We think, however, that it is a very simple thing to chalk or otherwise mark certain spots or lines upon a board, or on the floor, and instruct the patient how to try and touch the spots with accuracy with toe or heel or finger, and how to practise following the lines with foot or hand as he lies in bed, sits in a chair, or stands before the places marked upon the floor. These practises in co-ordinating the movements of the feet and legs, or hands and arms, are very easy to devise, and they have been recommended to out-patients at hospitals for years. We have before us a cardboard measuring 23 inches by 25 inches, which will fold in half, and upon this are certain simple diagrams, whose lines the patient is to be directed to follow accurately with his foot or hand. One-third of the booklet consists of directions as to the mode of training co-ordinate movements by means of them. We do not complain of this, because the idea is good; but it is the idea rather than the chart that is of value; as we have already said, a pencil and a piece of paper, or a piece of chalk with which to mark a floor, are all that are required, and we think that the price asked for the device before us is far more than it is worth.

Some of the Clinical Aspects of Pneumonia. By Donald W. C. Hood, C.V.O., M.D. (Cantab.), F.R.C.P. (Lond.), Senior Physician to the West London Hospital. With 5 charts in text 8vo. (London : John Bale, Sons, and Danielsson, Limited. 1907. Pp. 117. Price 5s. net.)

This excellent little brochure embodies the substance of clinical lectures and demonstrations delivered by the author at the West London Hospital. It therefore contains much that is not to be found in the text-books of medicine that are primarily intended for students. The common features of pneumonia are less touched upon than are the difficulties that arise in its diagnosis. The "acute abdomen" type, for example, in which it is at first uncertain whether the lesion is abdominal or thoracic; pneumonia with a cerebral type of onset; influenzal pneumonia and its variations; post-pneumonic empæmata and the difficulties they give rise to when between the lobes, or when not to be found on needling the chest; apical pneumonia, its anomalies, and its association with phthisis; pneumonia without physical signs; profuse hæmoptysis in pneumonia; and so forth. The author gives illustrative cases, and the book finishes with a chapter on the practical treatment of patients suffering from pneumonia of various degrees of complexity or extent. The letterpress is interesting and helpful. The medical practitioner will read and enjoy this little volume, and will find it full of practical and useful hints both as to the diagnosis and as to the treatment of cases of pneumonia that are not quite straightforward.

Hygiene of Nerves and Mind in Health and Disease.—By August Forel, M.D. Translated from the German by Austin Aikins, Ph.D. (London : John Murray, Albemarle Street, W. (The Progressive Science Series), 6s. net.)

To psychologists Dr. Forel's work on the hygiene of the nerves and mind is sufficiently well-known to need no introduction. To the general practitioner, however, this authorised English translation must be very welcome, since it gives, succinctly and clearly, the main points of interest in the book, omitting nothing that is material and adding nothing that is not wanted. A description and a detailed commentary of the book we do not purpose giving here. We have neither the space nor the intention to criticise all Dr. Forel's conclusions. Suffice it to say that they are all eminently practical, and that no one need be deterred, through any fear of wandering lost in the forests of German metaphysical speculation, from reading and enjoying his ably-written book, so excellently Englished by Dr. Aikins. Here and there one meets with expressions and turns which are not quite clear. The frequent use of new terms and new expressions—although, in justice to the author and translator, it must be said that an attempt is made to explain the significance of every new term used—is apt to worry the reader, especially when he finds that the new expression is but an old term in a novel dress. The introductory part of the book is admirably clear and concise, and on the whole we can thoroughly advise any practitioner, who desires to obtain a clear and condensed account of modern psychological developments, to read Dr. Forel's thesis and to add this volume to his library.

The Therapeutical Society's Transactions. Vol. V., 1907. (Demy 8vo., pp. 176. Figs. 12. Price 5s.)

This little volume is half as large again as was that issued by the Society last year; and, as usual, it contains the papers upon therapeutical subjects read and discussed before the Society during its last session. We say "last" advisedly, because the Therapeutical Society, during the five years of its existence, has grown to such important dimensions that it has now become attached to the Royal Society of Medicine, and will henceforth be the Therapeutical and Pharmacological Section of that Society.

All the papers in the volume before us are of interest; representing, as they do, the result of much work upon the value or otherwise of various forms of medicinal and other kinds of non-surgical treatment of disease, they cannot but be of practical value to all practitioners. We would instance, for example, Dr. Cecil Wall's paper upon the treatment of chorea by aceto-salicylic acid; the great advantage of this drug over arsenic in chorea is beginning to be more widely known, and we here have the original article upon the subject. Dr. W. E. Dixon's paper upon the mode of action of drugs suddenly awakens one to quite new ideas, and makes one begin to think that the old dictum about "drugs of which we know little . . . " is becoming inapplicable in the face of modern research and advance. Dr. George Oliver's paper upon the control of super-normal blood pressure contains very useful and practical information resulting from long-continued observations upon man.

We regret that we shall see no further volumes from this Society itself, but we look forward to the issue of equally interesting work by the Therapeutical Section of the Royal Society of Medicine.

The volume is obtainable only from the Honorary Secretary, 20 Hanover Square, W.

### SURGERY.

Some Points in the Surgical Anatomy of the Temporal Bone from Birth to Adult Life. By Arthur H. Cheatle, F.R.C.S., Aural Surgeon to King's College Hospital and to King Edward VII.'s Hospital for Officers. (London : J. and A. Churchill, Great Marlborough Street. 1907. Pp. ix + 131. Price 5s.)

This book comprises the Hunterian Lectures delivered by the author in 1906. In it he gives a description of the development of the various parts of the temporal bone from the time of infant life, laying particular stress on those facts which are of practical importance to the surgeon, as is particularly seen in the chapter on the variations in position of the lateral sinus. The feature of the book is the excellence of the illustrations. These are reproductions of photographs of the actual specimens exhibited at the College of Surgeons. They are quite admirable, and give the reader a clear idea of a very intricate portion of human anatomy. The subject is, of course, a special one, and the book will therefore appeal more particularly to all who are interested in, or directing their attention to, aural surgery.

Surgical Applied Anatomy. By Sir Frederick Treves, Bart., G.C.V.O., C.B., LL.D., F.R.C.S., Sergeant Surgeon-in-Ordinary to H.M. the King, Surgeon-in-Ordinary to H.R.H. the Prince of Wales, Consulting Surgeon to the London Hospital. Fifth edition, revised by Arthur Keith, M.D., F.R.C.S., Lecturer on and Senior Demonstrator of Anatomy at the London Hospital, Examiner in Anatomy at the Royal College of Surgeons of England. Pp. xxii and 640. Illustrations 107, of which 41 are in colour. (Cassell and Co., Limited, London, Paris, New York, Toronto and Melbourne. 1907. Price 9s.)

This book is so well known to the profession at large that it hardly needs any recommendation from us. The fact that it is now in its thirty-fourth thousand speaks for itself. Its popularity rests on a solid basis, for herein are set forth just those points in anatomy which are of the highest importance to the surgeon, with the reasons for their importance. This edition has been revised throughout by Mr. Keith. There could be no better guarantee that the work has been thoroughly done. The number of illustrations has been greatly increased, and many of them are now coloured. Some of the old figures have been withdrawn. As a natural result the matter has been increased, but not the bulk of the book, which is now printed on thinner paper. The text has been kept up to date, and the anatomy of the parts involved in modern surgical operations is described in full. As evidence of this we may instance fig. 26, which gives a good idea of the depth of the Gasserian ganglion from the surface and of its relations. The text would rather lead one to suppose that the operation described should be attributed to Rose instead of to Hartley and Krause, who first removed the ganglion by the temporal route. This, however, is a small matter, and does not detract from the general excellence of the book.

Diseases of the Male Generative Organs. By Edred M. Corner, F.R.C.S. (London: Henry Frowde, Oxford University Press, and Hodder and Stoughton, Warwick Square, E.C. 5s. net. Oxford University Manuals.)

We do not think it is the wish of the editors of the series to which this work belongs that the books shall replace the ordinary text-books used by students and practitioners. For the most part the volumes are small monographs of special subjects treated from a special point of view. In the majority of cases they have been excellently prepared. In this work, dealing with the disorders of the male generative organs, Mr. Corner has given a succint, yet clear and thoroughly sound, summary of such information as may be useful to the student, and there is indeed much to be found in his short chapters which one will not find in ordinary text-books such as the average student reads for examinations. The chapter on hydrocele, on the complications of varicocele operations, on ectopia and retentio testis, and on torsion of the testicle, are well worth reading, even by the practitioner and by the senior student. To a large extent the work is elementary—more so, perhaps, than the other works in the series—but the information given is thoroughly sound, details of operative work, which are more properly dealt with in larger works, are excluded, and special points in diagnosis and treatment are carefully considered. The work is one which reflects credit on the author—much more, indeed, than it does on the publishers, for printer's errors are, unfortunately, irritatingly plentiful. In a second edition these should be corrected. Mr. Corner might also take the opportunity of improving some of his sentences, which at present have a slovenly appearance, and give one the impression that they have been written in a hurry.

### SPECIAL SUBJECTS.

The Bacteriological Examination of Disinfectants. By William Partridge, F.I.C., with a preface by C. E. P. Fowler, D.P.H., F.R.C.S., Major, R.A.M.C. Pp. 66. (Published by the Sanitary Publishing Company, Limited. Price 2s. 6d. net.)

There has been much and rather heated discussion of late upon the question of how various disinfectants may best be standardised so that their comparative germicidal powers may be known with certainty. There is no ideal method of standardising them yet known, but there are many persons who hold that the "Rideal-Walker" process is the one which should be followed until a better is discovered. Unanimity upon the subject is certainly required, but that unanimity has not yet been attained. Some manufacturers standardise their disinfectants in one way, others in another, so that medical men who use the various preparations are liable to be misled as to the real effectiveness of each. The brochure before us is written in praise of the Rideal-Walker method of standardising disinfectants, about which we say something in another column. The author gives an interesting, if perhaps somewhat biassed, account of the advantages of this process over others, and he details the technique required. Medical men cannot but be interested in the controversy; but we think the book is more likely to be read by sanitary authorities, manufacturers of disinfectants, and by medical officers of health than by general practitioners.

Text-Book of Organic Chemistry for Medical Students. By Dr. G. v. Bunge. Translated with additions by R. H. A. Plimmer, D.Sc. Longmans, Green and Co., 39 Paternoster Row, London. 6s. net.

"It is quite impossible," remarks Professor von Bunge in his Preface to the English edition of his well-known work on organic chemistry, "for the student of medicine to find out for himself what is for him important and essential amongst the immense mass of chemical facts at his disposal. The necessity has therefore arisen for treating the rudiments of organic chemistry in a manner especially adapted to the requirements of medical students." With this object, the lectures which form the subject matter of the book have been written. As a text-book the work is admirable. The graphic representation of formula, which is throughout adopted, will be of the utmost help to the student, apart altogether from its mnemotechnic value, and the easy, flowing style which makes Professor Bunge's works so readable in the original German will help to render this volume popular with English students. Condensed as it is, the volume is yet an epitome of the essentials of organic chemistry, and should prove exceedingly useful to students preparing for their preliminary scientific examination. As editor and translator Dr. Plimmer has acquitted himself well. His translation is fluent and idiomatic, and his notes and additions are usually illuminative. To students of physiological chemistry the book should prove of real service.

